# Runx2 downregulates *Lpl* expression through super-silencer formation to alter lipid metabolism in Zhu Schwann cells after nerve injury

**DOI:** 10.1186/s11658-025-00796-6

**Published:** 2025-10-17

**Authors:** Zhaowei Zhu, Rui Kuang, Shouwen Su, Yujing Zhang, Guanggeng Wu, Yi Zhang, Vincent Pang, Xiang Zhou, Yan Yang, Ge Li, Bo He, Yangbin Xu

**Affiliations:** 1 Department of Plastic Surgery, The First Affiliated Hospital of Sun Yat-Sen University, No. 58 Zhongshan Road 2, Guangzhou, 510080 China; 2 Orthopaedic Trauma and Joint Department, Department of Orthopedics, The Third Affiliated Hospital of Sun Yat-Sen University, Guangzhou, 510000 China; 3 Department of Plastic Surgery, The First Hospital of China Medical University, No. 155 North Nanjing Street, Heping District, Shenyang, 110001 Liaoning Province China; 4 Department of Microsurgery, Trauma and Hand Surgery, The First Affiliated Hospital, Sun Yat-Sen University, Guangzhou, 510080 China; 5 Department of Dermatology, Guangzhou Dermatology Hospital, No. 56 Hengfu Road, Guangzhou, 510095 Guangdong China; 6 Guangdong Provincial Key Laboratory of Pathogenesis, Targeted Prevention and Treatment of Heart Disease, Medical Research Institute, Guangdong Provincial People’s Hospital (Guangdong Academy of Medical Sciences), Southern Medical University,, Guangzhou, 510100 China

**Keywords:** Runx2, Super-silencer, Zhu Schwann cells, He Schwann cells, Lipid metabolism

## Abstract

**Background:**

Phenotypic transformation of Schwann cells (SCs) plays a crucial role in nerve regeneration. Previous studies have demonstrated that *Runx2* significantly influences the biological behavior of SCs. Nonetheless, the regulatory mechanisms that govern its epigenetic regulation are not yet fully elucidated.

**Methods:**

To facilitate this investigation, an adenovirus for the overexpression of *Runx2* was constructed. Healthy adult Sprague–Dawley rats, weighing between 100 and 150 g and irrespective of sex, were randomly selected for the study. After establishing a model of sciatic nerve crush injury, tissue samples were harvested for histological analysis at both 4 and 7 days post-injury. In vitro, an *Runx2*-overexpressing SC line was established. Thorough analysis of transcriptome data, coupled with CUT&Tag sequencing of histones and transcription factors in SCs following *Runx2* overexpression, was conducted. Additionally, single-cell RNA sequencing data from GSE216665 were incorporated to elucidate the mechanistic role of *Runx2*. The findings were subsequently validated through dual-luciferase assays.

**Results:**

Following nerve crush injury, *Runx2*-positive SCs were identified at the injury site. Through comprehensive multiomics analysis, we discovered that lipid metabolism was disrupted in *Runx2*-overexpressing SCs. Further investigation established a detailed super-silencer landscape in these cells, revealing that elevated *Runx2* levels form a super-silencer within the transcriptional regulatory region of the *Lpl* gene, thereby downregulating *Lpl* expression.

**Conclusions:**

*Runx2* can modulate the biological behavior of SCs by forming super-silencers that interfere with the expression of lipid metabolism genes, such as *Lpl*, thereby altering the metabolic capacity of SCs.

**Graphical Abstract:**

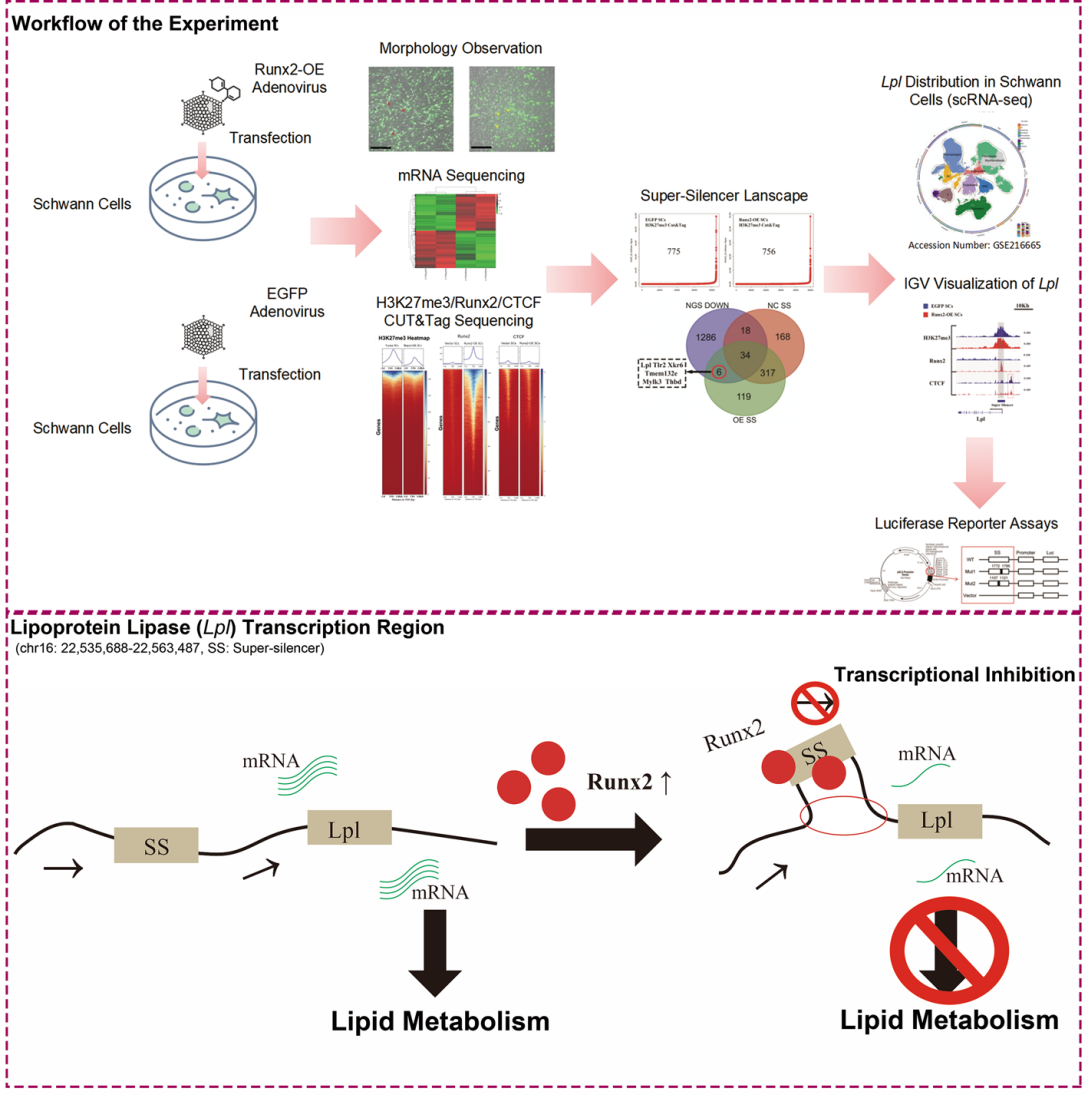

**Supplementary Information:**

The online version contains supplementary material available at 10.1186/s11658-025-00796-6.

## Highlights

1. We identified the first “super-silencer” landscape in SCs with Runx2 overexpression (Zhu1 SCs).
2. We validated that Runx2 suppresses *Lpl* expression in SCs through the formation of super-silencer sequences, regulating lipid metabolism in SCs during early peripheral nerve injury.
3. We describe modified molecular markers for repair-type SCs.

**Supplementary Information:**

The online version contains supplementary material available at 10.1186/s11658-025-00796-6.

## Background

The regeneration of tissues after injury requires the repair or replacement of damaged or lost cells. Some animals, such as salamanders and newts, can regenerate limbs and other organs after injury. This process involves reprogramming cells at the injury site into pluripotent stem cells or progenitor/immature cells with positional information, repairing lost cells or tissue structures by reproducing developmental processes [1]. In adult mammals, peripheral nerves are among the few tissues capable of extensive regeneration.

The regeneration of nerve axons after peripheral nerve injury repair is a continuous and complex process. SCs play a crucial role in nerve axon regeneration [2]. Following nerve injury, SCs undergo dedifferentiation and reprogramming, reverting to an active state similar to “immature SCs” in early developmental stages. Subsequently, these cells proliferate and reform myelin sheaths, ultimately completing the critical repair process of nerve regeneration. This plasticity mechanism enables SCs to effectively promote axonal regeneration and functional recovery after peripheral nervous system injury [3].

In our preliminary studies, we performed whole-transcriptome sequencing of sciatic nerves from Sprague Dawley (SD) rats at 4 and 7 days after crush injury, and found that the expression level of *Runx2* in nerves correlates with injury duration (database from Zhang (2021) [4]). Although some studies have attempted to explore the relationship between Runx2 and the state of SCs, there are currently only five publications on the role of Runx2 in peripheral nerves [3, 5–8].

Ding et al. discovered that Runx2 expression gradually increased after sciatic nerve crush, peaking at 1 week, suggesting that Runx2 may be involved in nerve axon growth and SCs differentiation and migration [5]. Wang et al. found that curcumin enhances SC proliferation and myelination through Runx2, promoting sciatic nerve injury repair [6]. Hu et al. also found that Runx2 promotes SC migration and myelin regeneration. These results all suggest that Runx2 plays a role in the process of SC dedifferentiation and reprogramming after peripheral nerve injury [7].

Through integrated multiomics analysis of Runx2-overexpressing (Runx2-OE) SCs combined with single-cell RNA sequencing (scRNA-seq) data from injured rat sciatic nerves, we discovered that Runx2 can promote phenotypic transformation of SCs by activating Sox2 [3]. Runx2 is highly expressed only in early repair-type Zhu1 SCs, while showing low to median expression in repair-type He SCs and late repair-type Zhu2 SCs. Sox2 is best known for its core role in maintaining embryonic stem cell self-renewal and pluripotency [9]. Research has also shown that it is one of the transcription factors that can help reprogram somatic cells into induced pluripotent stem cells [10, 11].

After SCs regain stem cell-like properties and become immature-like SCs, their biological functions inevitably change. To further investigate the epigenetic mechanisms by which Runx2 influences SCs, we established *Runx2*-OE SCs and conducted multiomics analysis. The results suggest that effects of Runx2 on cellular phenotype may not depend on histone modifications but rather directly target closed chromatin, affecting chromatin accessibility. Simultaneously, this influences the recruitment of other transcription factors, affecting the construction of key transcriptional elements, particularly influencing the status of negative regulatory factors, thereby influencing cellular functions [3].

To further explore how Runx2 affects cell fate through negative factors, this study aims to use histone modification (H3K27me3) chromatin accessibility using targeted endonuclease and tagmentation (CUT&Tag) and transcription factor (Runx2 and CTCF) CUT&Tag sequencing, combined with mRNA sequencing results from Runx2-OE SCs, to gain a deeper understanding of the epigenetic regulatory mechanisms of how Runx2 affecting SCs under injury conditions. The raw sequencing data were shared with those in He et al. [3].

## Methods

To investigate peripheral nerve regeneration mechanisms, adult Sprague–Dawley (SD) rats (100–150 g, mixed-sex cohort) were subjected to standardized sciatic nerve crush injury. Primary Schwann cells (SCs) were isolated from 3- to 5-day-old neonatal SD rats via enzymatic dissociation of sciatic nerves, followed by purification and expansion in vitro. All experimental protocols complied with the ethical guidelines of Sun Yat-Sen University’s Experimental Animal Administration Committee, incorporating perioperative analgesia, daily postoperative monitoring, and predefined humane endpoints. Complete details of all reagents (including manufacturers and dosages), instruments, and software are provided in Supplementary Tables S2–S3.

### Spatial–temporal profiling of Runx2 expression in crushed sciatic nerves via multiplex immunofluorescence

Adult Sprague–Dawley rats (100–150 g, both sexes, *n* = 3/group) were allocated into: normal controls (no surgery), 4-day post-crush (PI4d), and 7-day post-crush (PI7d) cohorts. The sciatic nerve crush model was established according to previous reports from literature [3, 4]. Focal sciatic nerve injury was induced by triple 30-s vascular clamp compressions at the piriformis muscle inferior border. Injured nerve segments were harvested at designated timepoints. Animals in the normal group underwent the same operation but without sciatic nerve clamp operation. The samples were processed and stained according to previous literature reports [3, 12]. Fixed 4% paraformaldehyde (PFA) samples underwent sequential immunofluorescence staining with anti-Runx2, NF200, CD31, and S100b. Nuclei were counterstained with 4′,6-diamidino-2-phenylindole (DAPI). Confocal imaging captured *z*-stacks from two randomized sections per sample.

Using ImageJ software, we measured the total fluorescence intensity of Runx2 in each field of view. The fluorescence intensity values were normalized by dividing the total Runx2 fluorescence intensity of each field by the average Runx2 fluorescence intensity of the control group, yielding relative fluorescence intensity values. GraphPad software was then used to analyze whether there were statistically significant differences in relative Runx2 intensity between groups and to generate statistical graphs. Since the data did not follow a normal distribution, the Kruskal–Wallis test was employed for analysis.

### Morphology changes of *Runx2*-overexpression SCs (Zhu1 SCs) in vitro

A recombinant adenoviral vector (Ad-*Runx2*-EGFP) expressing *Runx2* under cytomegalovirus (CMV) promoter was generated. Control vector expressing enhanced green fluorescent protein (EGFP) only (Ad-EGFP) was constructed in parallel.

In accordance with previous methods [3, 12], SCs from 3- to 5-day-old neonatal SD rats were isolated and purified. P0 SCs were expanded in DMEM/F12 + 10% fetal bovine serum (FBS) + 2 μM forskolin + 10 ng/mL neuregulin 1 (NRG1) at 37 °C/5% CO_2_. Cells at 90% confluence were dissociated for transduction. SCs transduced with Ad-*Runx2*-EGFP or Ad-EGFP (multiplicity of infection, MOI = 200) were subjected to imaging for 72 h using laser confocal microscopy. Morphometric parameters (cell area, elongation factor, and process complexity) were captured.

### mRNA profiling of *Runx2*-OE SCs

*Runx2*-OE and control (EGFP) SCs (*n* = 2 biologically independent replicates) underwent paired-end RNA sequencing by Shanghai Biotechnology Corporation [3]. Library preparation utilized the SMART-Seq^®^ HT kit, with quality control via Qubit 4.0 and Agilent 2100 Bioanalyzer. HISAT2 (version 2.0.4) aligned reads to Rnor_6.0 genome, followed by fragments per kilobase of transcript per million mapped reads (FPKM) normalization [13]. Differential expression was determined by edgeR (*p* < 0.05, |log_2_fold change (FC)|> 1). Gene set enrichment analysis identified enriched KEGG pathways.

### High-resolution epigenomic mapping of Runx2 regulatory landscapes

The CUT&Tag assay was performed as described previously with modifications [3, 14] and was performed by Guangzhou Huayin Health Medical Group Co., Ltd. (Guangdong, China). For H3K27me3, Runx2, and CTCF profiling, 1 × 10⁶ SCs underwent CUT&Tag following the manufacturer’s protocol with critical modifications. The raw sequencing data were shared with those in He et al. [3].

### Establishment and functional analysis of the super-silencer library

The rank ordering of super-enhancers (ROSE) algorithm was adapted to identify SSs by using H3K27me3 CUT&Tag data. The methodology has been described previously [15], and named ReSE. Specifically, the H3K27me3 peak files identified by MACS2 and the BAM files were used as inputs for the algorithm. Intergenic and intronic H3K27me3 peaks within 4 kb were stitched together to define a single silencer entity spanning a genomic region. Isolated peaks (without neighboring peaks within 12.5 kb) were considered individual silencers. Super-silencers (SSs) were defined as silencers with H3K27me3 intensity above a cutoff where the slope of the distribution plot of H3K27me3 intensity equals 1. The remaining silencers were classified as typical silencers (TSs). SSs and TSs were annotated using the annotate Peaks.pl tool from HOMER software (version 4.11). Motif enrichment analysis for SS and TS was performed using the findMotifsGenome.pl tool from HOMER software (version 4.11). A protein–protein interaction (PPI) network was constructed according to the intersection among SS in both groups and downregulated sequences from mRNA-seq.

### Dual-luciferase assay to verify the binding sites of the Runx2 peak region and *Lpl *SS

To elucidate the specific mechanism by which *Runx2* acts to inhibit *Lpl*, we employed the FIMO component of the MEME software suite to scan and identify Runx2 binding sites within the *Lpl* SS, with the detailed protocols reported in a previous study [3]. On the basis of the identified binding site sequences, we designed the following luciferase reporter constructs. The sequences of these reporters are listed in Supplementary Table S1.

*Lpl* SS region verification:

Wild-type (WT) vector: pGL3-promoter/WT;

Empty vector control: pGL3-promoter;

Mutant 1 vector: pGL3-promoter/Mut1 (1772-1786)

Mutant 2 vector: pGL3-promoter/Mut2 (1107-1121).

These reporter constructs were prepared as viral vectors and cotransfected into 293 T cells along with either the *Runx2* overexpression plasmid (*Runx2*-OE) or a control plasmid (pcDNA3.1) [3]. The luciferase reporter system was constructed by Dongzebio Co., Ltd. All results are presented as the mean ± standard error of the mean (SEM), with *n* = 4 plates of cells per condition.

### Spatial–temporal dynamics of *Lpl* in Schwann cell states

Leveraging a previously published sciatic nerve scRNA-seq atlas (GSE216665) [3, 16], we implemented a focused interrogation strategy for *Lpl* regulation: First, *Sox10 *^+^ *Plp1* ^+^ Schwann cells (*n* = 8421 cells) were isolated from integrated datasets (naïve, PI3d, PI12d, and PI60d) using Seurat’s subset function. For each cell type (Table 1), marker genes were defined as differentially expressed mRNAs (DEmRNAs) with an adjusted *p* value < 0.05 and a log_2_-fold change > 0.25 compared with the remaining cell types. For each group, DEmRNAs of each cell type were defined as genes with an adjusted *p* value < 0.05 and a log_2_-fold change > 0.25 between the two groups.

**Table 1:** Specific gene markers of different SCs (He’s classification) [3]

Nomination	Zhu	He	Remak	Myelinating/myelin*	HZ**
Marker	P75NTR	P75NTR	Egr2	Egr2	
	Bdnf	Mki67	Gfap	Mbp	
	Gdnf	Top2a	Cdh19	Mpz	
	Erbb3	Cdk1	Scn7a	Prx	
	Runx2***			Mag	
				Cldn19	

SCs are annotated on the basis of the expression of the marker genes *Sox10*, *Plp1*, and *S100b*

*Myelin SCs are defined as SCs that formed mature myelin in normal nerves or injured nerves, while myelinating SCs refer to SCs that are in the process of forming myelin sheaths but have not yet completed myelination

**HZ SCs indicate SC markers but do not belong to any other classification

***Zhu SCs include Zhu1 SCs and Zhu2 SCs, with Runx2 serving as a marker that distinguishes Zhu1 SCs from Zhu2 SCs

### Analysis of in-bulk tissue transcriptome data of nerve crush injury in SD rats

The transcriptomic sequencing data of SD rats’ sciatic nerve crush injury models were downloaded from the GEO database (accession no. GSE162548) [4]. The dataset includes samples from normal sciatic nerves (NC) and crushed nerves at 4 days (PI4d) and 7 days (PI7d) post-injury (*n* = 2). We conducted reanalysis of transcriptome data following previously reported methods [4, 17]. To compare gene expression levels among different genes and different samples, reads were converted into FPKM for normalization of gene expression levels. The Stringtie (version 1.3.0), (trimmed mean of M values (TMM) method and Perl scripts were used to calculate the FPKM value of each gene. The average expression values of *Lpl* at different injury times were obtained, and line graphs were drawn for intergroup comparison.

### Images, data processing, and statistical analysis

The data were processed by using SPSS and GraphPad Prism. One-way analysis of variance (ANOVA) with the Tukey multiple-comparison method was used to check the specific differences among groups. The chi-squared test was used to analyze percentage data, setting the test standard *α* as 0.05.

The Shapiro‒Wilk test was used to analyze the luciferase reporter system, setting *p* > 0.05. The Brown–Forsythe test was used to judge whether homogeneity of variances was met or not (*p* > 0.05). Finally, corrected one-way ANOVA, followed by Dunnett’s test for pairwise comparisons, was performed. The vector or WT group as control was used and compared with the mutant groups.

## Results

### Expression of Runx2 after nerve injury and analysis of differentially expressed genes in *Runx2*-OE SCs

**Fig. 1:**
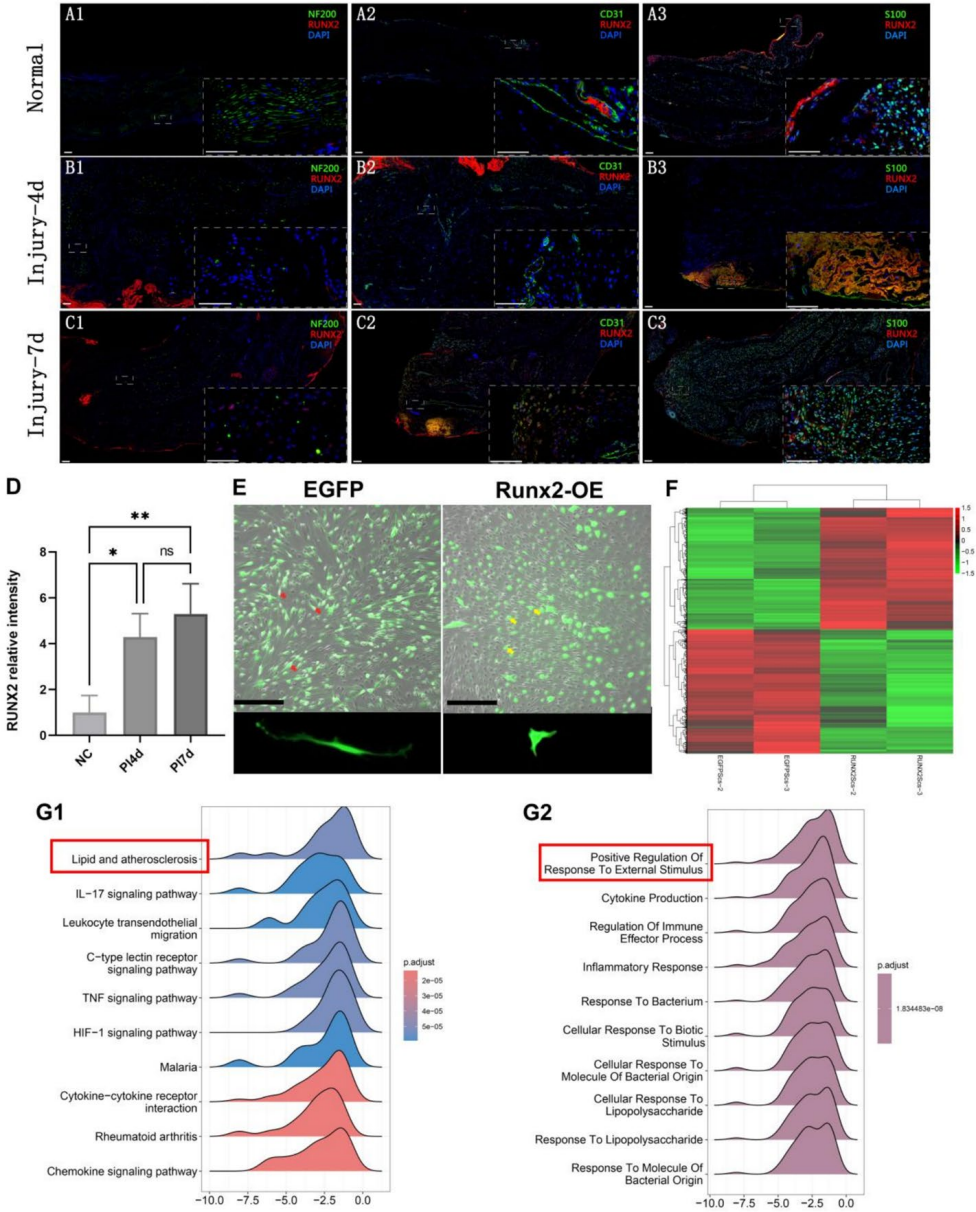
Histological observation of Runx2 expression in SD rats following sciatic nerve crush injury at 4 and 7 days, and transcriptome analysis of SCs after *Runx2*-overexpression (*Runx2*-OE). **A**–**C** Immunofluorescence images of peripheral nerves in normal nerves (scale bars represent 100 μm, **A**) and at 4 days (**B**) and 7 days (**C**) post-injury (*n* = 3). In all panels, green represents NF200 ^+ ^axons (green) and Runx2 ^+^ (red) in 1, CD31 ^+^ endothelial cells (green) and Runx2 ^+^ (red) in 2, and S100 ^+^ SCs (green) and Runx2 ^+^ (red) in 3. Blue denotes nuclei (DAPI ^+^). **D** Bar chart depicting quantitative analysis of Runx2 immunofluorescence staining in peripheral nerves at different time points post-injury. **E** Morphological observation of SCs after *Runx2*-OE. Red arrows in EGFP group point to control SCs, while yellow arrows in *Runx2*-OE group point to SCs after *Runx2* overexpression. Scale bars represent 50 μm. **F** Heatmap showing the distribution of differentially expressed mRNAs (DEmRNAs) between the two groups. Red indicates upregulation, and green indicates downregulation, *n* = 2. **G** Ridge plot showing GSEA analysis of DEmRNAs, with red boxes highlighting significantly enriched pathways, the color gradient from red to blue indicates increasing *p* values. **G1** KEGG enrichment analysis, **G2** GO enrichment analysis. *p < 0.05, **p < 0.01, ns indicates no significance

Immunofluorescence staining for CD31/NF200/S100 and Runx2 was performed at the injury site at different time points after peripheral nerve crush injury. The results revealed a significant increase in Runx2 expression in SCs during the early phase of injury, with noticeable coexpression in the S100-positive areas, which were predominantly localized in SCs (Fig. 1a–c). Quantitative analysis of Runx2 immunofluorescence staining revealed that the ratios for PI4d and PI7d groups were significantly elevated compared with the normal group (*p* < 0.05), but no statistically significant difference was observed between PI4d and PI7d groups (Fig. 1d).

The morphologies of these in vitro-cultured cells were examined using light microscopy. It could be found that SCs in the EGFP group (designated as He SCs) exhibited characteristic bipolar elongated morphology, whereas SCs in the *Runx2*-OE group (designated as Zhu SCs) displayed a rounded cell shape with short protrusions (Fig. 1e).

Smart-seq mRNA sequencing analysis identified 1317 upregulated and 1345 downregulated genes in *Runx2*-OE SCs, with notable upregulation of *Runx2*, *Notch*, *Mki67*, and *Pten* and downregulation of *Il1rl1*, *Lpl*, and *Egfr*, among others (Fig. 1f). GSEA analysis revealed negative correlations between *Runx2*-OE SCs and KEGG pathways related to lipid and atherosclerosis (normalized enrichment score, NES = −2.10, *p* < 0.001) and hypoxia-inducible factor (HIF)−1 signaling pathway (NES = −2.16, *p* < 0.001), while GO terms were associated with positive regulation of response to external stimulus (NES = −2.27, *p* < 0.001) and inflammatory response (NES = −2.34, *p* < 0.001) (Fig. 1G1, G2), suggesting that *Runx2* might influence the biological properties of SCs by altering cellular lipid metabolism and immune responses.

### Analysis of key regulatory transcription factors Runx2 and CTCF in Runx2-OE SCs

**Fig. 2:**
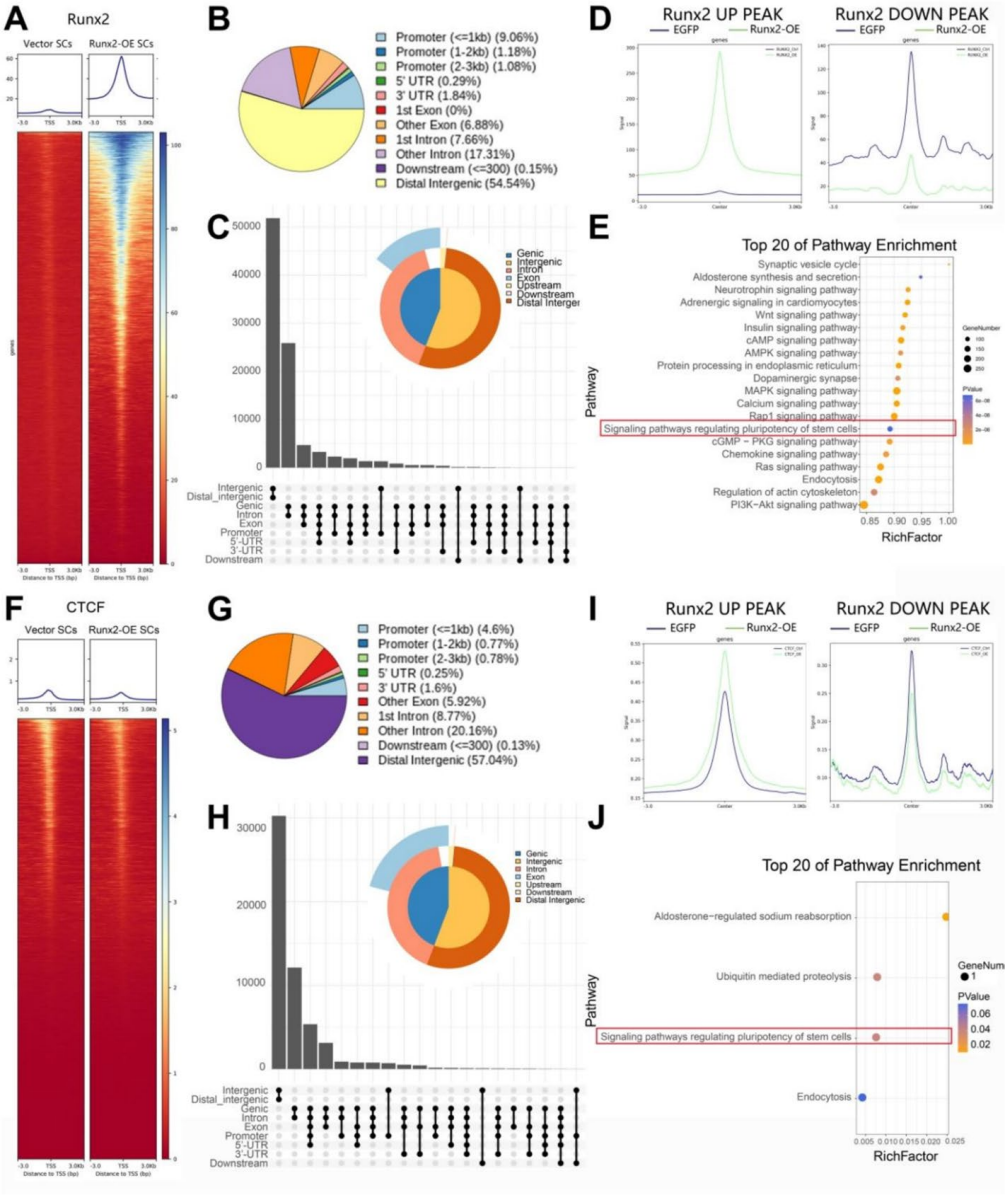
Runx2 and CTCF Cut&Tag sequencing in *Runx2*-OE transfected SCs. **A**–**E** Distribution and number of Runx2 peaks in *Runx2*-OE SCs. **A** Heatmap showing the distance between Runx2 peaks and transcription start sites (TSS) after *Runx2*-OE. **B** Pie chart illustrating the feature distribution of Runx2 peaks in the *Runx2*-OE and vector groups; **C** Upset plot visualizing feature distribution of Runx2 peaks. **D** Comparative analysis of the distribution of Runx2 signals in the *Runx2*-OE group versus the EGFP control group, centered on the upregulated/downregulated Runx2 CUT&Tag peaks (±3 kb). The purple symbol denotes the control group (EGFP SCs), whereas the green symbol represents the *Runx2*-OE group. **E** List of KEGG enrichment pathways showing genes with Runx2 peaks in *Runx2*-OE SCs. **F**–**J** Distribution and number of CTCF peaks in *Runx2*-OE SCs. **F** Heatmap showing the distance between CTCF peaks and TSS after *Runx2*-OE. **G** Pie chart illustrating the feature distribution of CTCF peaks in the *Runx2*-OE and vector groups; **H** Upset plot visualizing feature distribution of CTCF peaks. **I** Comparative analysis of the distribution of CTCF signals in the *Runx2*-OE group versus the EGFP control group, centered on the upregulated/downregulated Runx2 CUT&Tag peaks (±3 kb). The purple symbol denotes the control group (EGFP SCs), whereas the green symbol represents the *Runx2*-OE group. **J** List of KEGG enrichment pathways showing genes with CTCF peaks in *Runx2*-OE SCs. In **E** and **J**, the circle size represents the number of genes, and the color gradient from red to blue indicates increasing *p* values. The red boxes indicate key pathways

Further CUT&Tag-seq analysis was performed to examine Runx2 and CTCF binding capacity in *Runx2*-OE SCs. In Runx2 CUT&Tag-seq results, peaks in the *Runx2*-OE group were primarily concentrated in the Transcription Start Site (TSS) region (Fig. 2a), with 11.32% located in the promoter region (Fig. 2b); the corresponding Upset plot is shown in Fig. 2c. Runx2 signals were significantly higher in the OE group compared with the control group within *Runx2*-upregulated peak regions, while showing an opposite pattern in *Runx2*-downregulated peak regions (Fig. 2d). The upregulated peaks in the *Runx2*-OE group were mainly enriched in pathways including synaptic vesicle cycle, neurotrophin signaling pathway, WNT signaling pathway, cAMP signaling pathway, signaling pathway regulating pluripotency of stem cells, and PI3K–AKT signaling pathway (Fig. 2e).

In CTCF CUT&Tag-seq analysis, no significant difference was observed in the number of peaks at TSS regions between OE and control groups (Fig. 2f), with 6.15% located in the promoter region in the OE group (Fig. 2g); the corresponding Upset plot is shown in Fig. 2h). CTCF signals were notably higher in the OE group within Runx2-upregulated peak regions, while showing an opposite pattern in Runx2-downregulated peak regions, suggesting potential significant interactions between Runx2 and CTCF in the former peak regions (Fig. 2i). The upregulated peaks in the *Runx2*-OE group were primarily enriched in pathways including aldosterone-regulated sodium reabsorption, ubiquitin mediated proteolysis, and signaling pathway regulating pluripotency of stem cells (Fig. 2j).

### Changes in chromatin H3K27me3 modification status in *Runx2*-OE SCs

**Fig. 3:**
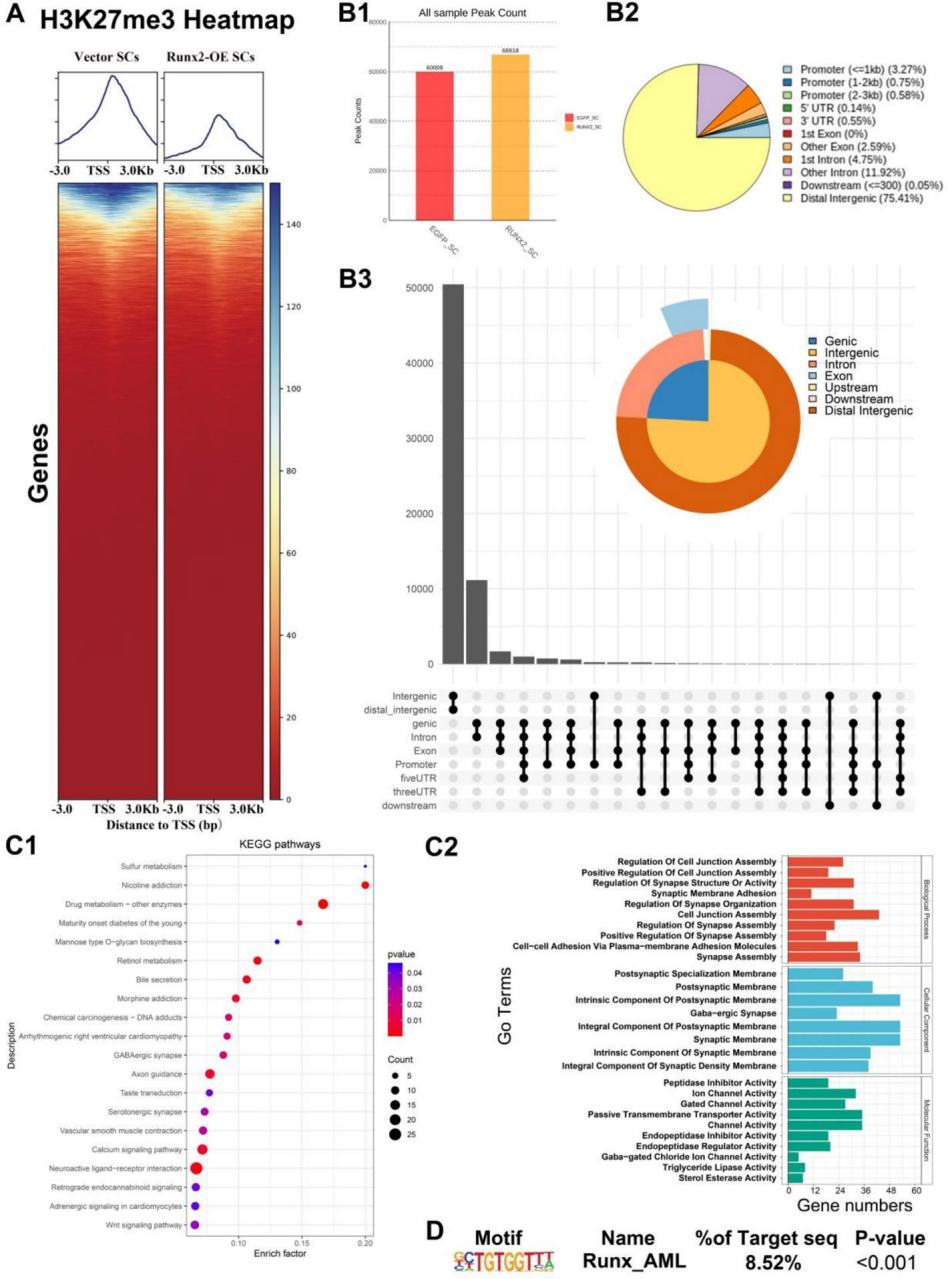
H3K27me3 Cut&Tag sequencing in *Runx2*-OE transfected SCs. **A** Heatmap showing the distance between H3K27me3 peaks and transcription start sites (TSS) after *Runx2* overexpression. **B** Distribution and number of H3K27me3 peaks in *Runx2*-OE SCs. **B1** Bar graph showing differences in H3K27me3 peak numbers between different groups; **B2** Pie chart illustrating the feature distribution of H3K27me3 peaks in the *Runx2*-OE and vector groups; **B3** Upset plot visualizing feature distribution of H3K27me3 peaks. **C** List of functional enrichment pathways showing genes with H3K27me3 peaks in *Runx2*-OE SCs. **C1** KEGG pathway analysis. The circle size represents the number of genes, and the color gradient from red to blue indicates increasing *p* values. **C2** GO analysis. **D** HOMER-predicted motifs responsive to *Runx2*-OE H3K27me3 data

To better understand the histone methylation modifications in SCs following *Runx2*-OE, we performed H3K27me3 CUT&Tag-seq analysis (Fig. 3a). The results indicated that *Runx2*-OE cells exhibited more peaks compared with the control group (vector) (66,918 versus 60,009; Fig. 3B1), while the vector group showed greater peak accumulation in the transcription start site (TSS) region than the *Runx2*-OE group (Fig. 3a). In *Runx2*-OE cells, peaks were predominantly concentrated in distal intergenic regions (75.41%), with 4.6% located in promoter sequences (Fig. 3B2); the corresponding Upset plot is shown in Fig. 3B3.

Enrichment analysis of target genes corresponding to signals in *Runx2*-OE revealed that KEGG pathways were mainly concentrated in sulfur metabolism, axon guidance, serotonergic synapse, calcium signaling pathway, and neuroactive ligand-receptor interaction (Fig. 3C1). GO analysis suggested that these signals were primarily enriched in processes regulating cell connections and synaptic assembly, as well as lipid metabolism processes such as triglyceride lipase and sterol esterase activity (Fig. 3C2). HOMER motif scanning of *Runx2*-OE SCs revealed potential binding of Runx_AML (8.52%, Fig. 3d).

### Establishment of Runx2-related super-silencer landscape in *Runx2*-OE SCs

**Fig. 4:**
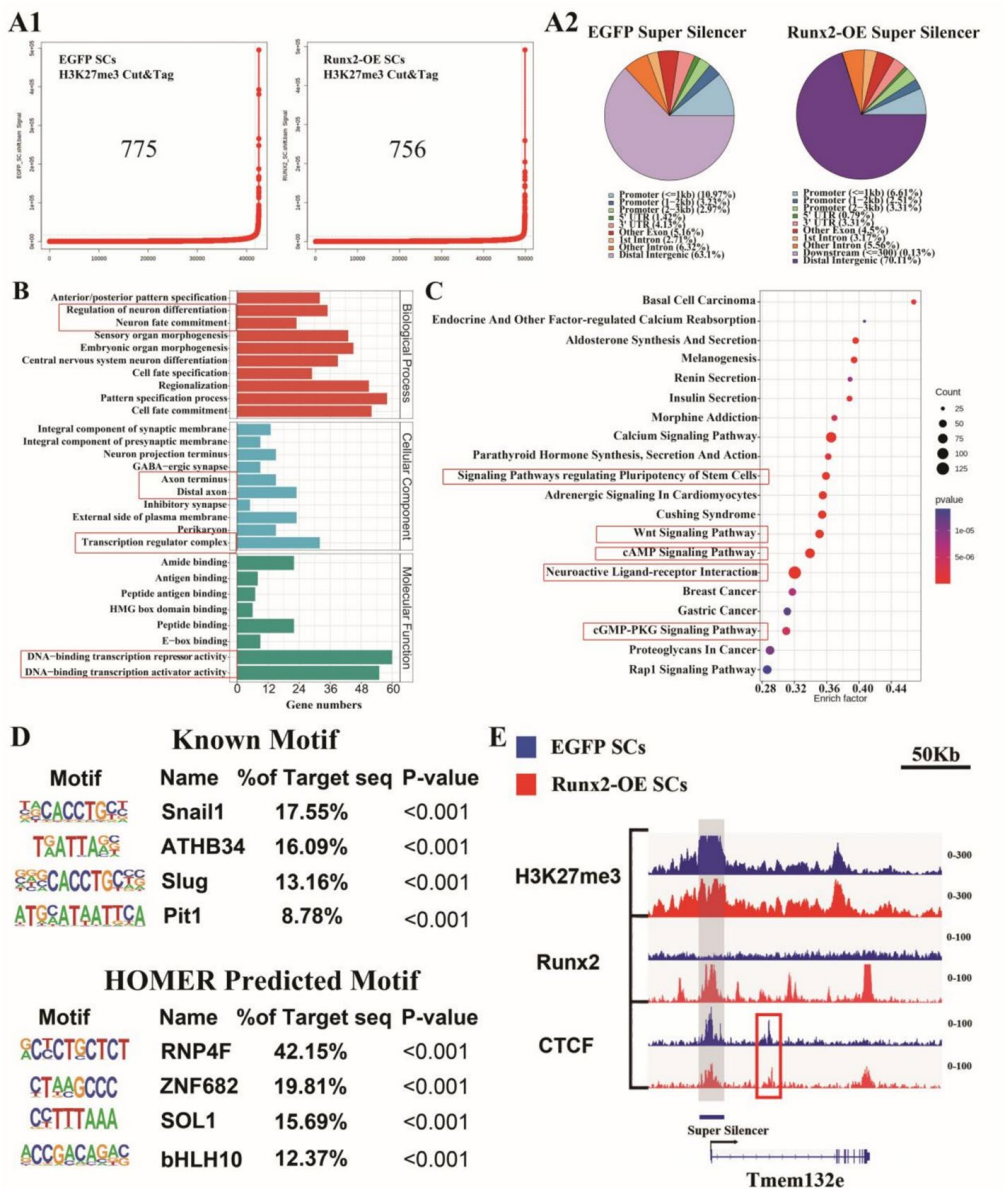
Super-silencer (SS) landscape construction in* Runx2*-OE transfected SCs. **A** Distribution of SS region in *Runx2*-OE SCs. **A1** Hotkey plot of SS sorted by the ROSE algorithm in *Runx2*-OE and EGFP groups; **A2** Pie chart illustrating the feature distribution of SS in the *Runx2*-OE and EGFP groups. **B** List of GO enrichment pathways showing genes with SS in *Runx2*-OE SCs. The red boxes indicate key pathways. **C** List of KEGG enrichment pathways showing genes with SS in *Runx2*-OE SCs. The red boxes indicate key pathways. The circle size represents the number of genes, and the color gradient from red to blue indicates increasing *p* values. **D** HOMER-predicted motifs responsive to *Runx2*-OE SS. **E** Visualization of the *Tmem132e* gene showing SS, with the grey box indicating key elements (E) within the SS, and the red box indicating CTCF peaks area

Following Cai’s methodology [15], we performed ReSE analysis on the H3K27me3 CUT&Tag results from both groups of cells. We identified 775 methylation-enriched sites in the control group (EGFP SCs) and 756 of these sites in the *Runx2*-OE group (Fig. 4A1). In accordance with Cai et al.’s definition, we considered these sites as SSs. In the control group, 17.17% of these SSs were located around promoters, whereas in *Runx2*-OE SCs, this proportion was 12.43% (Fig. 4A2).

GO and KEGG functional enrichment analyses of these genes revealed that, in *Runx2*-OE cells, genes associated with SS were involved in biological functions such as the regulation of neuronal differentiation, synaptic assembly, motor organ morphology, and cell fate determination. These genes were localized in presynaptic/postsynaptic membranes and axon terminals, influencing the activity of transcriptional regulatory complexes and DNA-binding transcriptional repressor/activator molecules (Fig. 4b). KEGG analysis suggested that these genes could affect SCs pluripotency pathways, the WNT signalling pathway, the cAMP signalling pathway, and neuroactive ligand‒receptor interactions (Fig. 4c).

HOMER scanning of motifs binding in SS regions revealed that the known motifs mainly included Snail1 (17.55%) and ATHB34 (16.09%), while the predicted binding motifs were RNF4F (42.15%), ZNF682 (19.81%), Sol1 (15.69%), and bHLH10 (12.37%). Other motifs are shown in Fig. 4d. Visualization of the regulatory regions of *Tmem132e* (Fig. 4e) genes using Integrative Genomics Viewer (IGV) software revealed SS regions around the coding sequences. These regions contained increased Runx2 signals, along with enriched H3K27me3 peaks.

### Runx2 downregulates lipoprotein lipase (*Lpl*) expression through SS formation, interfering with Schwann cell lipid metabolism

**Fig. 5:**
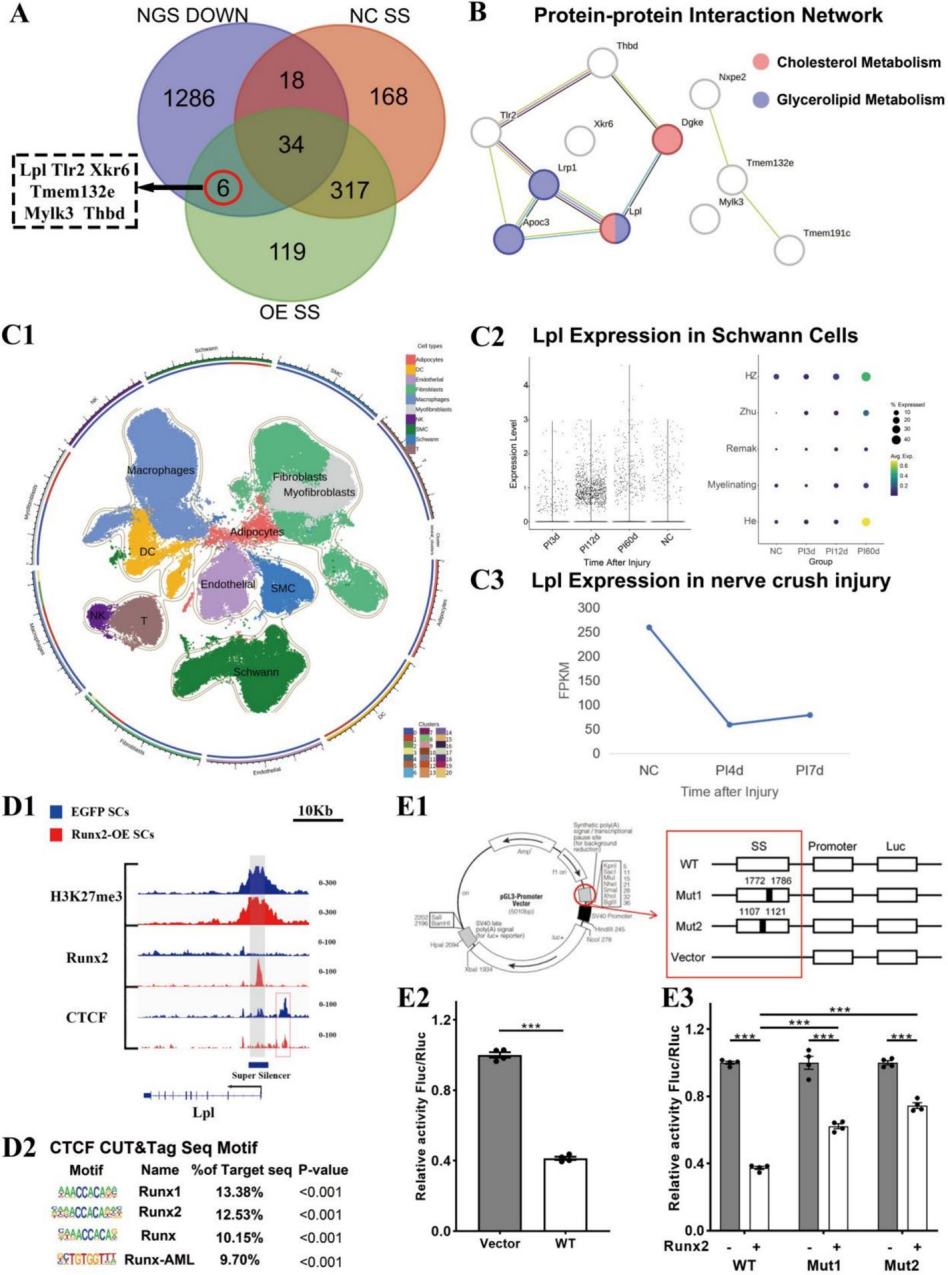
Super-silencer landscape construction in* Runx2-*OE transfected SCs. **A** Intersection diagram of SS target sequences uniquely present in *Runx2*-OE SCs and downregulated in transcriptome data, with arrows indicating genes influenced by Runx2-related SS. **B** Protein–protein interaction (PPI) network of target genes of Runx2-related SS. **C** Single-cell RNA sequencing (scRNA-seq) and bulk tissue transcriptome analysis of SD rats at different time periods after nerve crush injury. The scRNA-seq data includes 3 days (PI3d), 12 days (PI12d), and 60 days (PI60d) post-injury compared with those of normal nerves (NC). Data source: GSE216665. The bulk tissue transcriptome data includes normal nerves (NC), 4 days post-injury (PI4d), and 7 days (PI7d) post-injury data. Data source: GSE162548. **C1** UMAP distribution plot of different cell types in scRNA-seq data; **C2** Violin plot and bubble plot illustrating the expression changes of *Lpl* in SCs during different injury time. Yellow, green, and purple indicate gradually decreasing gene expression compared with the mean. The bubble size represents the proportion of cells expressing the gene within each cell type, with larger areas indicating higher expression proportions. **C3** Line graph illustrating the expression changes of *Lpl* in the injury segment at different time points after peripheral nerve injury. **D** Visualization of *Lpl* gene. **D1** IGV graph of the *Lpl* gene showing SS, with the grey box indicating key elements (E) within the SS and the red box indicating CTCF peaks area. **D2** HOMER-predicted motifs responsive to CTCF CUT&Tag-seq in *Runx2*-OE SCs; **E** Luciferase reporter assay to investigate the mechanism by which Runx2 downregulates *Lpl* expression through the formation of a super-silencer (SS) (*n* = 4). **E1** Schematic of the luciferase reporter plasmid construction and the *Lpl*-SS gene sequence (reused from He et al. [3]); **E2** Bar graph showing the luminescence response in 293 T cells for the *Lpl*-SS wild type (WT) and vector control group; **E3** Bar graph comparing the luminescence response in 293 T cells for mutant versions of the *Lpl*-SS region (Mut1 and Mut2) and the wild type (WT) before and after overexpression of *Runx2*; *p < 0.05, **p < 0.01, ***p < 0.005

Intersection analysis of SS solely existing in *Runx2*-OE group and downregulated sequences from mRNA-seq revealed six genes that presented downregulated expression and SS formation following *Runx2*-OE. These genes were *Lpl*, *Tlr2*, *Tmem132e*, *Mylk3*, *Xkr6*, and *Thbd* (Fig. 5a). The construction of a PPI network with these genes showed that *Lpl* could form an interaction network with *Thbd* and *Tlr2*. Moreover, *Lpl* was associated with cellular cholesterol metabolism and glycolipid metabolism processes, suggesting that changes in *Lpl* expression would affect SC function through altering lipid metabolism (Fig. 5b). Meanwhile, Thbd and Tlr2 are two molecules with different functions, participating in the coagulation–anticoagulation system and innate immune response processes, respectively.

We reanalyzed previously published bulk tissue transcriptome data (GSE162548) from SD rat sciatic nerve crush injury at 4 days (PI4d) and 7 days (PI7d) [4] and found that *Lpl* expression decreased in the early post-injury period (PI4d) and remained lower than normal nerves (NC) at PI7d, indicating that *Lpl* expression is suppressed in the early stages of peripheral nerve injury (Fig. 5C3). To observe the distribution and function of *Lpl* in peripheral nerve injury, we reanalyzed the GSE216665 dataset. After annotation, the cell clusters in GSE216665 could be divided into ten cell populations, as shown in Fig. 5C1. Analysis of *Lpl* expression in SC clusters at different time points after injury revealed that *Lpl* expression began to increase in SCs primarily at 12 days post-injury (PI12d) and continued through 60 days post-injury (PI60d) (Fig. 5C2). Further observation of *Lpl* changes in different SCs subtypes showed that the *Lpl* gene was mainly expressed in He and HZ SCs at PI60d (Fig. 5C1).

Visualization of the regulatory regions of *Lpl* (Fig. 5D2) using IGV software revealed SS regions around the coding sequences. This region contained enriched H3K27me3 and Runx2 peaks in the promoter area. Meanwhile, some CTCF signals could be found around the SS region in these genes whether *Runx2*-OE or not. Further HOMER scanning of CTCF CUT&Tag for motif analysis revealed that Runt family-related transcription factors, including Runx1, Runx2, Runx, and Runnx-AML, showed high occurrence frequencies (9.70–13.38%, as shown in Fig. 5D2).

To verify the existence of *Lpl* SS after *Runx2*-OE, we employed a dual-luciferase reporter assay. Different luciferase reporter plasmids were cotransfected with either the *Runx2*-OE plasmid or the control plasmid (pcDNA3.1) into 293 T cells (Fig. 5E1). Statistical analysis of the luciferase reporter system data via the Shapiro‒Wilk test indicated that the data satisfied the assumption of normality (*p* > 0.05). However, the Brown–Forsythe test suggested that the assumption of homogeneity of variances was not met (*p* > 0.05). The results revealed that the fluorescence intensity in the wild-type (WT) group was significantly lower than that in the empty vector group, suggesting that *Runx2* can bind to the selected *Lpl* SS fragment and inhibit *Lpl* gene expression (Fig. 5E2).

Corrected one-way ANOVA of the WT, Mut1, and Mut2 groups revealed an *F* statistic of 185.4 with a corrected *P* < 0.0001. Pairwise comparisons revealed statistically significant differences between the WT and Mut1 groups and between the WT and Mut2 groups (*P* < 0.0001). Upon *Runx2* overexpression, the WT group presented a marked decrease in fluorescence intensity compared with that in the control group, indicating a strong inhibitory interaction between *Runx2* and the *Lpl* SS region in the WT construct. Interestingly, when the predicted binding sites within the SS were mutated, both the Mut1 and Mut2 groups presented reduced fluorescence intensity compared with their respective controls following *Runx2* overexpression. However, this reduction was less pronounced than that in the WT group, suggesting that Mut1 and Mut2 retain some capacity to interact with *Runx2*, albeit with diminished efficacy (Fig. 5E3).

These findings further corroborate that *Runx2* suppresses *Lpl* expression by promoting the formation of an SS in the *Lpl* transcriptional regulatory region and binding to this SS. Moreover, through IGV observation of *Tmem132e* and *Lpl* (Figs. 4e and 5D1), high CTCF signals were detected near the SS regions of target genes, but these signal peaks showed little change before and after *Runx2*-OE. We hypothesize that, after *Runx2*-OE, the three-dimensional structure of the SS regulatory region is altered, resulting in shortened distances between CTCF and regulatory regions, further suppressing the expression of target genes.

## Discussion

The Runt-related transcription factor (Runx) family includes Runx1, Runx2, and Runx3, all sharing a highly homologous Runt-associated DNA-binding domain [3]. Among them, Runx2 (also termed Osf2/Cbfa1, Pebp2αA, or AML-3) acts as a transcription factor critically involved in tumorigenesis, metastasis, and invasion [18], as well as in osteogenic and chondrogenic differentiation/maturation [19]. It also modulates adipogenic and epithelial differentiation processes in adipose-derived stem cells [20]. Owens et al. demonstrated that *Runx2* overexpression induces epithelial–mesenchymal transition (EMT) and suppresses differentiation in mammary cells, whereas *Runx2* knockout inhibits breast cancer cell invasion/migration and improves survival rates [21], underscoring its central role in cellular proliferation and differentiation. Notably, Fang et al. [22] identified Runx2 as a master regulator of fibrotic gene expression in pulmonary fibrosis. Conditional *Runx2* knockout reduces pathological fibroblast activation, suppresses extracellular matrix (ECM) deposition, and mitigates fibrotic progression, highlighting its pivotal role in alveolar fibroblast phenotypic switching [22]. In the central nervous system (CNS), Runx2 is predominantly expressed in gliomas and astrocytes [23, 24]. Tiwari et al. further revealed that Runx2 regulates astrocyte differentiation and promotes cellular maturation [24]. Kalinski et al. performed scRNA-seq on mouse sciatic nerve injury models and found that, 3 days post-injury, SCs mainly divided into three types: SC1, SC2, and SC3. Among these, SC3 highly expresses transcription regulators including *Sox4*, *Runx2*, *Hmga1*, *Jun*, and the POU family member *Pou3f1* [25], but they did not conduct further research on *Runx2*.

### Runx2 influences nerve injury repair by regulating SC phenotype and metabolic processes

Ding et al. found that Runx2 could stimulate SC differentiation and enhance SC proliferation and migration ability through activating the Akt/GSk3β pathway, ultimately promoting nerve regeneration [5]. Hu et al. demonstrated using a SC-specific Runx2 knockout mouse model with sciatic nerve injury that Runx2 deletion impaired SC migration and caused myelination deficits [7]. While this study established that Runx2 is essential for SCs remyelination, it did not comprehensively elucidate its regulatory mechanisms, particularly within epigenetic contexts. Hung et al. performed chromatin immunoprecipitation sequencing (ChIP-seq) analysis on rat peripheral nerve injury models and found that activated c-Jun binds to *Runx2* enhancer sequences, inducing Runx2 upregulation and participating in regulation of SC activation and myelin breakdown and reorganization [8].

We classified reprogrammed SCs post-injury into three subtypes: He SCs (proliferative SCs), HZ SCs (transition SCs), and Zhu SCs (promyelinating-like SCs). We found that Runx2 expression was first detected in Zhu SCs, corresponding to the earliest appearance of Zhu1 SCs in the pseudotime trajectory. Subsequently, Runx2 expression in SCs gradually decreased as He SCs emerged. By day 60, Runx2 expression in Zhu SCs had declined to levels comparable to other SC subtypes. Eventually, all SCs differentiated into either myelin SCs or Remak SCs [3]. This manifestation indicates that Runx2 is an important molecule in maintaining the phenotype of Zhu1 SCs, or in other words, Runx2 is a marker that distinguishes Zhu1 SCs (Runx2 positive) from Zhu2 SCs (Runx2 negative).

In this experiment, we found extensive overlap between SCs marker S100 and Runx2 staining regions. Additionally, 72 h after infecting rat primary SCs with *Runx2* overexpression adenovirus (*Runx2*-OE) and corresponding control virus (EGFP), *Runx2*-OE SCs showed notably flatter cell bodies compared with controls, with retracted processes at both ends and disappearance of intercellular network structures. Smart-seq mRNA sequencing analysis of differentially expressed genes revealed that genes differentially expressed in *Runx2*-OE SCs were mainly related to lipid metabolism and inflammatory response, while key transcription factor (Runx2 and CTCF) target genes were enriched in signaling pathways regulating pluripotency of stem cells. Furthermore, inhibitory histone modification signals (H3K27me3) after OE were enriched in regulatory regions of lipid metabolism-related target genes, further suggesting that Runx2 might influence SC fate by regulating cellular lipid metabolism processes (Table 1).

### Runx2 can downregulate lipoprotein lipase (*Lpl*) in SCs through the formation of a super-silencer

There are usually two different regions in gene sequences, namely activation regions and suppression regions, which are jointly involved in regulating the expression of cell identity-related genes [15]. Activated regions associated with cell self-renewal are often regulated by super-enhancers (SEs). SEs are regions enriched in H3K27ac or transcription factors, which can greatly increase the expression of target genes [26]. Conversely, cell lineage-specific genes are often associated with repressive transcription elements, particularly in the CpG regions of chromosomal structure [27]. CpG regions are often enriched in H3K27me3 and the catalytic group of the PRC2 complex [28]. Huang et al. reported that regions enriched in H3K27me3 act as silencers, which could function through the connection between the H3K27me3-DNaseI high-response region and gene expression [29]. Cai et al. defined regions enriched in H3K27me3 as an SS via methods that search for SEs. They also reported that, unlike conventional silencers, SSs interact with chromatin through loop formation, inhibiting the function of long segments of chromatin [15]. CUT&Tag sequencing analysis of H3K27me3 in Runx2-OE cells indicated that Runx2 affects the proportion of genes undergoing histone modification.

In accordance with Cai’s method [15], we investigated the results of CUT&Tag sequencing analysis of H3K27me3 in two groups of cells via ReSE analysis and found 756 SSs in SCs after *Runx2* upregulation. The proportion of SSs around the promoter was 17.17% in EGFP cells and 12.43% in *Runx2*-OE SCs. GO enrichment and KEGG pathway analyses revealed that the genes with SSs after *Runx2* overexpression participate in synapse assembly, motor organ morphology, cell fate, and other biological functions. These genes are expressed in the presynaptic/postsynaptic membrane and affect the function of SCs through the stem cell pluripotency pathway, the WNT signaling pathway, the cAMP signaling pathway, and neuroactive ligand‒receptor interactions. Considering that SS primarily functions by affecting gene expression, we performed intersection analysis between SS that only appeared in *Runx2*-OE cells and downregulated genes after OE, ultimately establishing a *Runx2*-dependent super-silencer landscape in SCs. This landscape primarily includes six genes: *Lpl*, *Tlr2*, *Tmem132e*, *Mylk3*, *Xkr6*, and *Thbd*. Through expanded PPI network analysis, we found that *Lpl*, *Thbd*, and *Tlr2* could form an interaction network. Moreover, *Lpl* is connected to both cellular cholesterol metabolism and glycolipid metabolism processes, suggesting that changes in *Lpl* expression would alter SC functional states by affecting lipid metabolism.

### In the early stage of peripheral nerve injury, Runx2 reduces *Lpl* expression in Zhu1 SCs, affecting lipid metabolism

Visualization of the *Lpl* gene revealed the presence of SS regions in its transcription zone, and certain regions (1772–1786 and 1107–1121) were found to bind with Runx2 to induce a response. After the overall SS region and these two segments were knocked out, the inhibitory effect of Runx2 on *Lpl* expression was significantly weakened, further confirming that *Runx2* can interfere with *Lpl* expression by forming a SS.

Studies have shown that myelin lipid metabolism can provide energy for starved axons, highlighting the critical role of lipid metabolism in the nervous system [30, 31]. Lipoprotein lipase (LPL), a key hydrolase, plays a central role in lipid metabolism and energy homeostasis [32]. Rothe et al. demonstrated that LDL receptor-mediated pathways internalize intraneural lipoproteins into SCs and peripheral neurons, repurposing cholesterol/cholesterol esters (CEs) from myelin degradation for membrane biosynthesis during remyelination and axonal regrowth. Following peripheral nerve injury, the breakdown of axons and myelin sheaths releases large amounts of cholesterol, CEs, and sphingomyelin. Therefore, during the early phase of injury, SCs need to focus on processing these lipids, rather than exogenous lipids [33]. For this reason, The expression of *Lpl*, which processes exogenous lipids, decreases in Zhu1 SCs during the early stage of injury.

In the later phase of peripheral nerve injury, when degraded myelin-derived lipids are depleted, exogenous lipid supplementation becomes necessary. Huey et al. proposed that LPL-mediated hydrolysis of exogenous triglycerides (TGs) serves as a major source of free fatty acids (FFAs) for SCs, likely playing a pivotal role in peripheral nervous system myelination. As a TG-specific hydrolase, LPL catabolizes TGs within lipoproteins (e.g., chylomicrons, very low-density lipoprotein(VLDL)) to generate FFAs and monoacylglycerols, thereby regulating local fatty acid availability to support late-stage myelin biosynthesis and repair [34, 35]. Therefore, *Lpl* expression increases in SCs during the late stage of injury.

In summary, after peripheral nerve injury, Zhu1 SCs, with high expression of *Runx2*, preferentially utilize cholesterol/CEs derived from myelin breakdown during the early phase by downregulating *Lpl* expression to minimize exogenous lipid utilization. By inhibiting the expression of *Lpl*, Runx2 can weaken the ability of SCs to metabolize lipids, promoting Zhu1 SCs to better adapt to the characteristics of lipid metabolism after engulfing disintegrated myelin sheaths in the early stage of injury.

### Runx2 binding to SS may induce three-dimensional structural changes in target gene regulatory regions, downregulating target genes through CTCF-assisted regulation

From the visualization of *Tmem132e* and *Lpl* (Figs. 4E and 5D1), we observed high CTCF signals near the SS regions of target genes. These signals showed little change in peak values before and after *Runx2*-OE. We hypothesized that, after Runx2 binds to SS, it may alter the three-dimensional structure of the regulatory region, leading to shortening distances between CTCF and regulatory regions, thereby assisting in the suppression of target gene expression.

CCCTC-binding factor (CTCF) is a nuclear DNA-binding protein first discovered by Lobanenkov et al. in 1990. CTCF negatively regulates chicken c-myc expression through interactions with three regularly spaced repeats of the CCCTC DNA motif [36]. CTCF is a multivalent DNA-binding protein containing 11 zinc fingers and is universally expressed in most vertebrate tissues [37]. CTCF can bind to tens of thousands of conserved DNA sites, with over 30% located in intronic regions and over 50% in intergenic regions [38]. Conventional CTCF target sites are highly conserved and mainly located in intergenic regions, while cell type-specific sites are primarily found in introns [39]. CTCF has also been identified as an RNA-binding protein, with CTCF–RNA interactions involving CTCF dimerization, long-range genomic binding sites, chromatin looping, and gene regulation [40]. CTCF can also regulate various post-translational modification processes by affecting transcription factors, chromatin remodeling factors, methylation regulators, histone modification factors, and splicing factors [41]. Owing to its unique role in chromatin architecture, CTCF is known as the “genome architect,” capable of regulating the expression of various molecules and thereby affecting their epigenetic functions [42]. CTCF can act as a transcription factor directly activating or suppressing target gene expression, or as a chromatin insulator indirectly interfering with enhancer/silencer–promoter contacts [43]. Furthermore, it can regulate gene expression by mediating chromatin looping and altering the spatial distance between genomic sites [44–46].

However, these hypotheses regarding whether CTCF plays a role and whether there is a synergistic effect between its function and SS still require more experimental validation, such as CTCF knockout experiments and chromosome conformation capture (3C) or high-throughput chromosome conformation capture (HiC) experiments in *Runx2*-OE SCs to examine chromatin structure. If hypotheses are confirmed, they would provide deeper insights into the epigenetic regulatory mechanisms in SC metabolism and enable precise regulation of SC functions in response to age and environmental changes in the future, promoting rapid SC response after nerve injury and timely adjustment to facilitate rapid peripheral nerve regeneration.

## Conclusions

In the early stage after peripheral nerve injury, Runx2 is upregulated in SCs, causing the phenotypic conversion of SCs to Zhu1 SCs, and establishes a super-silencer (SS) within the transcriptional regulatory region of *Lpl*, leading to the downregulation of *Lpl* expression.

## Supplementary Information

Additional file 1

## Data Availability

The data that support the findings of this study are available from the corresponding author upon reasonable request. Transcriptomic and CUT&Tag sequencing data GSE271351, GSE271353, and GSE271356 series records are available at: https://www.ncbi.nlm.nih.gov/geo/query/acc.cgi?acc=GSE271351. https://www.ncbi.nlm.nih.gov/geo/query/acc.cgi?acc=GSE271353. https://www.ncbi.nlm.nih.gov/geo/query/acc.cgi?acc=GSE271356.

## Abbreviations

SC: Schwann cell
scRNA-seq: Single-cell RNA sequencing
SD: Sprague-Dawley
SE: Super-enhancers
SS: Super-silencers
TS: Typical silencers
PPI: Protein–protein interaction
*Runx2*-OE: *Runx2* overexpression
CTCF: CCCTC-binding factor
GO: Gene Ontology
KEGG: Kyoto Encyclopedia of Genes and Genomes
ROSE: Rank ordering of super-enhancers
FPKM: Fragments per kilobase of transcript per million mapped reads
DEmRNAs: Differentially expressed mRNAs
FC: Fold change
PCA: Principal component analysis
CCA: Canonical correlation analysis
UMAP: Uniform manifold approximation and projection
ANOVA: One-way analysis of variance
TSS: Transcription start site
IGV: Integrative Genomics Viewer
WT: Wild-type
*Lpl*: Lipoprotein lipase
CUT&Tag: Chromatin accessibility using targeted endonuclease and tagmentation
ChIP-seq: Chromatin immunoprecipitation sequencing
EMT: Epithelial–mesenchymal transition
ECM: Extracellular matrix
CNS: Central nervous system
CEs: Cholesterol esters
TGs: Triglycerides
FFAs: Free fatty acids
VLDL: Very low-density lipoprotein
TMM: Trimmed mean of M values
