# Supplementary material for: Runx2 downregulates Lpl expression through super-silencer formation to alter lipid metabolism in Zhu Schwann cells after nerve injury
Source: Cell Mol Biol Lett. 2025 Oct 17;30:120. doi: 10.1186/s11658-025-00796-6 (PMC12535136; doi:10.1186/s11658-025-00796-6)
Supplement: Supplementary file 1 — Additional file 1 [file 11658_2025_796_MOESM1_ESM.docx]

**Supplementary information**

**Table S1.** Luciferase reporter sequence of *Lpl.* The bold and underlined positions in the sequence are the mutation areas.

| Site | Length (bp) | Sequence |
| --- | --- | --- |
| WT  -Promoter | 3971 | GGTGGGGTTAGATAGAGGGGACTGTTCTGCTGTCAGAAAAGTAGCAGCACTGAAGTAACAGGTGACACTGAAGACGGGATGAACGCCATCAGGGAAGGAGCACCGAGTGTCTGAAGACTCAGAGGAACTCAGCTACCGTCTCAGATAacacacacacacacacacacacacacatcacaATGCAGCCTTGGTGATGACAATGTGGAAACAGGCTGTGCCCAGAGGAAAGGGGACAATGACCGGACTGGACCATCTGACAGACAGAAGCTGTCAGCTGAGTATAACAGCCCGCTCTATAACGCTGTGGGCTAAAGGGAAGCTGTGTTAAAAATACACTCTGTCAAACACACGGAACATCTTTCCACCGAGATTCTTCCCCCGCTTGTCCCCCACACCTTTCAATGCCGTTATTAATCAATCACTCCCATGTCGAGCTCTTTTCCAGGTATTTAACTCTGATATTAGCCTGCAAGTGAGCACTGTGGCTGAACAGGACTTCATGCAGCCGGACCctctatgctccctctcttagctctctgctttcctgcCTGGGGCCAGGAAAGGCTGCAGGGGCTCCAGGAAGAAGCCCAGGGCAATGTCTCCCAAACTCATACACTCTTCGTGGAAAAAAAAGAAAAACATCAAGAGTGAACAAGGAAACTGGGAGCCTAGAGGGGAGCTGGGTTCACTCCAAGCTGTGCCTTTCACAGTCTCACCCTAGGTGAATTCATTTGTGGGACAAAGGGCTTGGCCTGCTGTTGTAGAAGAAGGAAAGTGCTAGTAGAGAGAACAGAACGTCGATAAGAGGAAATCATTCCACATCTGCTTAGGAATAATATTCCTCCAATACATTTTATTGATAGGAAAATCAGGTGTTGGCTAAAATCCAAGATTATTATGTAATTACTTAGGTGTCCCTTTAAACCAAACTATAAGGGAGGGGCCCCAGGGCACTTTGAGTCTTGCCAACTTTCCTAAGAGCTCATGCGGCTAAACCCAGAAGGGTGACACTAAGCAGCTGAAAAGCTGGGTCCAGAGCTTCTGAAGGAAGCAAAACCTGAAAATGGAGACGACCTTTTCCACGGGAGTTTACGGTAAAAGTCGTG**TTATACTGTGGTGTA**GTTTCTGAAATATGAATTGAGCGTTTGCTCCTATTTTCTTAACCAGAGTTGCAAACTATGTAGAAAGATAGCTTGCCACAGCAGAGACAGAATCCCCAGTGTCCTCAAAACACCCGAGATTCTTACTTTAGTGCAGTTCTGCCATGACCGATTCTCTCGCCTTTGATAACAAGACATTTACAACTATACGCAGAAATAAAAGCTAGCTTTCAGCTGCACTCATAGACTCCACTAAAACTGGAATTAGGCTGCGGACGTGCCACCTTAGAGCACCTCCAGGGGACACCACCCTGGTGGCGAGTCCACCAGCAGCAACACAAGGAAACTCCAGCCTCCTTTTATGGCTGAGTTCAGTCATTTTTTTGTGTCTGTGTTCTGTTAGCTCAATCAAGGTTCAAGTAACAAGGCTTCTCAATTGTGGGCAGGTCTGGACTTCAAGGCCATAAAGTAACTTGCTACCGACCCTAGTCGTCTTCTCTAGGCAGAGAGCAGGCAGAGCCGTGGAGATGACCGATTTTGGGACCATAATGGTGAAAGCGACCTCCTAACTGCTAACTGCTAACTGCTCTAGAAAATCCCATGTGCTCTCAGCTAGGGCGAGCCTCTCCTAGGACAGCAAGGATAGGACACAGTGCTGAACTGGCAACCTC**CAAGCCACAGGTTCC**AGTCTGCTGATGGCTGAGCCTCGTTTTCTGGAAGATAGGCTCTTTGCTGGGCTTTCCTGAATACCAAGCTAAGCCGAGCGAGCGCAAGGAACGCTTACAGGACGCGGTTTCCCAGGACTCCGTGCCATGCGTTCCCCGGCACCGCCGCGCATTCCCACTCTCACAGTAGCCCATAGCCGAGAGCGACAGTGCCCTATGCAGTTGGAATGCACACAACGCGTCCTCCCACCCGCACCCGCGATGGGGGCACACTCGGTTCACTCTTCCCGCCTACAGAGGGGTCTGACCCGTAGTCCCAGCAAAGTTAGGGTCACCTTGGCCCACATGCCTTGCATTCAGCAGCCCCACTGCCTATGAGGAGGTTACCACCGATCACTACAGCTTCTCTCCACCTCTGcgcccccacccccatcccacgcgtcacacccccgcccccgccGTTCCCTGTGATGGCCGGCGGCGGGAGAGAGCTGGGGACGGAGCGGGAAATCGGGagaatgaaaaaaataaagaaagaaagaaagaagaagaagaagCGGGAACCCGGAGTCGCCACAGCGTGATGGGAGGCAGAGTCTCTCCTTCCTCCTCCTGGATTCCACTCCAGCCACCCTTCCCGGGAATCTCTTTGGCTATTGCGCCTGCTTAGTGCACGTCCTTTCCAACTTTCTCCCTCCCATCTTCAGATGGAGAAAAAGTGGTCTGGTCCCGGTCTGCAGGTGGAGGGGAGCTGGCGCGAAAAACATACCGTCTGCTGCGGCCACCCCTCCGCGGAAGGCGGTCAAACTCTGGAGCCAAACTCCCAGGGCCACCAGGAGCAGGGCTTTGCTCTCCATCTCCGGGCGCGCCCTTCTGCTTTGCTGCTGGAACTGCGCTGCGCGGGCAGTCAGGCGGGCGGGGATCCGCGTTGCAAAGGGTTGAGCCGGAGGCGTCAGAGGACTAGAGCGCGGTGGAGCGCCGCGGGGCAAGTCAACCTTTAAAGGGGAGGGCAGAACAGCTACAAGGGGCAGAATTTCTTGGAGGAGGAGGAATCGAGTCTGACACCGTTTCCACTGCACAGCTGTTTAAGTGACTGGAAATATGCAAATAAGCCTCATCACCTATTGGCTATAAAATCATAAGTTGGGGGGAGGACTCTAAATTCACTTCCAAATATTTGAGCACACTGTAACGAGGCTCAACGGTGCCAGCGAGAAGAAGGGGAAAGGGCAGGAGGGAAAGCTTGCTTGTCGTTTAGGCTCCAGTTTGCCTATCCAGAGGAACCTGCTCCCCAGGGACCCCTGTAGAGGCCGGGAGGAGGGGTTTACGCTGATTTATGTTAAGTAGTCTACGATCAGTTTTCTGAGGGGAAGAAGTTTTGCTCTTAAACAGCGCGTGTGTCCAACACTTTCACCCCCTCCCTTTTGATAAATCTTTGTTAAACTTGAAAGATCCTTTCAAACGTACCCCCGCCACACACACACATACAGGTCCCCACGGGCCAAAGGGACCTTAGGACACATATTGCTCAGCGCCTGGTGCCTGCACGGTCTGCCTTTTATCGTTGGCAATATGGATCTCCTTCTGTGGTAGAAGAAAGGAAATTAGTTAATAATTCTAATTTTTACCTTATGTAAATTGTACTCGATCCTCTAATTCAGTAAGAAACTCAAGAATAACATCTATTTTCAGAGGCAGAAAGTCATGGTCAAATATTGTAAACTATCCTGGACTTTCAGTGGGAGAAGGCATGCCAAATTACCAATCCAAACTTACAAAGACAGTACATAGATTAGCGATGTTCCAGGGTGAGGGGGATATGGGGCGCTGGGAGTGTGGGGGCAGGGTGAGGATCTTGATGGAAATATCCCGAATTCCACTAGGCTTGTCATTGTATGACTGTGTAGATGCTTTAATATTAAATTGTGCTCTGCCAGGGTTGAGACTGAGCTTTTGCAAAGTGGATTAGCACATGTTTGTAGAAAAGCAAGAAAAATAAAATCACATCGTATACTGTGCTCATCTGCAAGTATCTTTTTAAATGAAGAAGGTGTTGATTAGCTAGAGAAAACCATAAAGATTAAATTCCCCACCTTAATGCTGAGAAGCCGTCTCAAAAAATCAGTTCTCTTTACTGATCTCAGTATTTTTAATATGGTCTCCTCTTcacacacacacacacacacacacacacacacacacacac |
| Mut1  -Promoter  (1772-1786) | 3971 | GGTGGGGTTAGATAGAGGGGACTGTTCTGCTGTCAGAAAAGTAGCAGCACTGAAGTAACAGGTGACACTGAAGACGGGATGAACGCCATCAGGGAAGGAGCACCGAGTGTCTGAAGACTCAGAGGAACTCAGCTACCGTCTCAGATAacacacacacacacacacacacacacatcacaATGCAGCCTTGGTGATGACAATGTGGAAACAGGCTGTGCCCAGAGGAAAGGGGACAATGACCGGACTGGACCATCTGACAGACAGAAGCTGTCAGCTGAGTATAACAGCCCGCTCTATAACGCTGTGGGCTAAAGGGAAGCTGTGTTAAAAATACACTCTGTCAAACACACGGAACATCTTTCCACCGAGATTCTTCCCCCGCTTGTCCCCCACACCTTTCAATGCCGTTATTAATCAATCACTCCCATGTCGAGCTCTTTTCCAGGTATTTAACTCTGATATTAGCCTGCAAGTGAGCACTGTGGCTGAACAGGACTTCATGCAGCCGGACCctctatgctccctctcttagctctctgctttcctgcCTGGGGCCAGGAAAGGCTGCAGGGGCTCCAGGAAGAAGCCCAGGGCAATGTCTCCCAAACTCATACACTCTTCGTGGAAAAAAAAGAAAAACATCAAGAGTGAACAAGGAAACTGGGAGCCTAGAGGGGAGCTGGGTTCACTCCAAGCTGTGCCTTTCACAGTCTCACCCTAGGTGAATTCATTTGTGGGACAAAGGGCTTGGCCTGCTGTTGTAGAAGAAGGAAAGTGCTAGTAGAGAGAACAGAACGTCGATAAGAGGAAATCATTCCACATCTGCTTAGGAATAATATTCCTCCAATACATTTTATTGATAGGAAAATCAGGTGTTGGCTAAAATCCAAGATTATTATGTAATTACTTAGGTGTCCCTTTAAACCAAACTATAAGGGAGGGGCCCCAGGGCACTTTGAGTCTTGCCAACTTTCCTAAGAGCTCATGCGGCTAAACCCAGAAGGGTGACACTAAGCAGCTGAAAAGCTGGGTCCAGAGCTTCTGAAGGAAGCAAAACCTGAAAATGGAGACGACCTTTTCCACGGGAGTTTACGGTAAAAGTCGTG**TTATACTGTGGTGTA**GTTTCTGAAATATGAATTGAGCGTTTGCTCCTATTTTCTTAACCAGAGTTGCAAACTATGTAGAAAGATAGCTTGCCACAGCAGAGACAGAATCCCCAGTGTCCTCAAAACACCCGAGATTCTTACTTTAGTGCAGTTCTGCCATGACCGATTCTCTCGCCTTTGATAACAAGACATTTACAACTATACGCAGAAATAAAAGCTAGCTTTCAGCTGCACTCATAGACTCCACTAAAACTGGAATTAGGCTGCGGACGTGCCACCTTAGAGCACCTCCAGGGGACACCACCCTGGTGGCGAGTCCACCAGCAGCAACACAAGGAAACTCCAGCCTCCTTTTATGGCTGAGTTCAGTCATTTTTTTGTGTCTGTGTTCTGTTAGCTCAATCAAGGTTCAAGTAACAAGGCTTCTCAATTGTGGGCAGGTCTGGACTTCAAGGCCATAAAGTAACTTGCTACCGACCCTAGTCGTCTTCTCTAGGCAGAGAGCAGGCAGAGCCGTGGAGATGACCGATTTTGGGACCATAATGGTGAAAGCGACCTCCTAACTGCTAACTGCTAACTGCTCTAGAAAATCCCATGTGCTCTCAGCTAGGGCGAGCCTCTCCTAGGACAGCAAGGATAGGACACAGTGCTGAACTGGCAACCTC***TGGATTGTGAACCTT***AGTCTGCTGATGGCTGAGCCTCGTTTTCTGGAAGATAGGCTCTTTGCTGGGCTTTCCTGAATACCAAGCTAAGCCGAGCGAGCGCAAGGAACGCTTACAGGACGCGGTTTCCCAGGACTCCGTGCCATGCGTTCCCCGGCACCGCCGCGCATTCCCACTCTCACAGTAGCCCATAGCCGAGAGCGACAGTGCCCTATGCAGTTGGAATGCACACAACGCGTCCTCCCACCCGCACCCGCGATGGGGGCACACTCGGTTCACTCTTCCCGCCTACAGAGGGGTCTGACCCGTAGTCCCAGCAAAGTTAGGGTCACCTTGGCCCACATGCCTTGCATTCAGCAGCCCCACTGCCTATGAGGAGGTTACCACCGATCACTACAGCTTCTCTCCACCTCTGcgcccccacccccatcccacgcgtcacacccccgcccccgccGTTCCCTGTGATGGCCGGCGGCGGGAGAGAGCTGGGGACGGAGCGGGAAATCGGGagaatgaaaaaaataaagaaagaaagaaagaagaagaagaagCGGGAACCCGGAGTCGCCACAGCGTGATGGGAGGCAGAGTCTCTCCTTCCTCCTCCTGGATTCCACTCCAGCCACCCTTCCCGGGAATCTCTTTGGCTATTGCGCCTGCTTAGTGCACGTCCTTTCCAACTTTCTCCCTCCCATCTTCAGATGGAGAAAAAGTGGTCTGGTCCCGGTCTGCAGGTGGAGGGGAGCTGGCGCGAAAAACATACCGTCTGCTGCGGCCACCCCTCCGCGGAAGGCGGTCAAACTCTGGAGCCAAACTCCCAGGGCCACCAGGAGCAGGGCTTTGCTCTCCATCTCCGGGCGCGCCCTTCTGCTTTGCTGCTGGAACTGCGCTGCGCGGGCAGTCAGGCGGGCGGGGATCCGCGTTGCAAAGGGTTGAGCCGGAGGCGTCAGAGGACTAGAGCGCGGTGGAGCGCCGCGGGGCAAGTCAACCTTTAAAGGGGAGGGCAGAACAGCTACAAGGGGCAGAATTTCTTGGAGGAGGAGGAATCGAGTCTGACACCGTTTCCACTGCACAGCTGTTTAAGTGACTGGAAATATGCAAATAAGCCTCATCACCTATTGGCTATAAAATCATAAGTTGGGGGGAGGACTCTAAATTCACTTCCAAATATTTGAGCACACTGTAACGAGGCTCAACGGTGCCAGCGAGAAGAAGGGGAAAGGGCAGGAGGGAAAGCTTGCTTGTCGTTTAGGCTCCAGTTTGCCTATCCAGAGGAACCTGCTCCCCAGGGACCCCTGTAGAGGCCGGGAGGAGGGGTTTACGCTGATTTATGTTAAGTAGTCTACGATCAGTTTTCTGAGGGGAAGAAGTTTTGCTCTTAAACAGCGCGTGTGTCCAACACTTTCACCCCCTCCCTTTTGATAAATCTTTGTTAAACTTGAAAGATCCTTTCAAACGTACCCCCGCCACACACACACATACAGGTCCCCACGGGCCAAAGGGACCTTAGGACACATATTGCTCAGCGCCTGGTGCCTGCACGGTCTGCCTTTTATCGTTGGCAATATGGATCTCCTTCTGTGGTAGAAGAAAGGAAATTAGTTAATAATTCTAATTTTTACCTTATGTAAATTGTACTCGATCCTCTAATTCAGTAAGAAACTCAAGAATAACATCTATTTTCAGAGGCAGAAAGTCATGGTCAAATATTGTAAACTATCCTGGACTTTCAGTGGGAGAAGGCATGCCAAATTACCAATCCAAACTTACAAAGACAGTACATAGATTAGCGATGTTCCAGGGTGAGGGGGATATGGGGCGCTGGGAGTGTGGGGGCAGGGTGAGGATCTTGATGGAAATATCCCGAATTCCACTAGGCTTGTCATTGTATGACTGTGTAGATGCTTTAATATTAAATTGTGCTCTGCCAGGGTTGAGACTGAGCTTTTGCAAAGTGGATTAGCACATGTTTGTAGAAAAGCAAGAAAAATAAAATCACATCGTATACTGTGCTCATCTGCAAGTATCTTTTTAAATGAAGAAGGTGTTGATTAGCTAGAGAAAACCATAAAGATTAAATTCCCCACCTTAATGCTGAGAAGCCGTCTCAAAAAATCAGTTCTCTTTACTGATCTCAGTATTTTTAATATGGTCTCCTCTTcacacacacacacacacacacacacacacacacacacac |
| Mut2  -Promoter  (1107-1121) | 3971 | GGTGGGGTTAGATAGAGGGGACTGTTCTGCTGTCAGAAAAGTAGCAGCACTGAAGTAACAGGTGACACTGAAGACGGGATGAACGCCATCAGGGAAGGAGCACCGAGTGTCTGAAGACTCAGAGGAACTCAGCTACCGTCTCAGATAacacacacacacacacacacacacacatcacaATGCAGCCTTGGTGATGACAATGTGGAAACAGGCTGTGCCCAGAGGAAAGGGGACAATGACCGGACTGGACCATCTGACAGACAGAAGCTGTCAGCTGAGTATAACAGCCCGCTCTATAACGCTGTGGGCTAAAGGGAAGCTGTGTTAAAAATACACTCTGTCAAACACACGGAACATCTTTCCACCGAGATTCTTCCCCCGCTTGTCCCCCACACCTTTCAATGCCGTTATTAATCAATCACTCCCATGTCGAGCTCTTTTCCAGGTATTTAACTCTGATATTAGCCTGCAAGTGAGCACTGTGGCTGAACAGGACTTCATGCAGCCGGACCctctatgctccctctcttagctctctgctttcctgcCTGGGGCCAGGAAAGGCTGCAGGGGCTCCAGGAAGAAGCCCAGGGCAATGTCTCCCAAACTCATACACTCTTCGTGGAAAAAAAAGAAAAACATCAAGAGTGAACAAGGAAACTGGGAGCCTAGAGGGGAGCTGGGTTCACTCCAAGCTGTGCCTTTCACAGTCTCACCCTAGGTGAATTCATTTGTGGGACAAAGGGCTTGGCCTGCTGTTGTAGAAGAAGGAAAGTGCTAGTAGAGAGAACAGAACGTCGATAAGAGGAAATCATTCCACATCTGCTTAGGAATAATATTCCTCCAATACATTTTATTGATAGGAAAATCAGGTGTTGGCTAAAATCCAAGATTATTATGTAATTACTTAGGTGTCCCTTTAAACCAAACTATAAGGGAGGGGCCCCAGGGCACTTTGAGTCTTGCCAACTTTCCTAAGAGCTCATGCGGCTAAACCCAGAAGGGTGACACTAAGCAGCTGAAAAGCTGGGTCCAGAGCTTCTGAAGGAAGCAAAACCTGAAAATGGAGACGACCTTTTCCACGGGAGTTTACGGTAAAAGTCGTG***CCGCGTCACAACACG***GTTTCTGAAATATGAATTGAGCGTTTGCTCCTATTTTCTTAACCAGAGTTGCAAACTATGTAGAAAGATAGCTTGCCACAGCAGAGACAGAATCCCCAGTGTCCTCAAAACACCCGAGATTCTTACTTTAGTGCAGTTCTGCCATGACCGATTCTCTCGCCTTTGATAACAAGACATTTACAACTATACGCAGAAATAAAAGCTAGCTTTCAGCTGCACTCATAGACTCCACTAAAACTGGAATTAGGCTGCGGACGTGCCACCTTAGAGCACCTCCAGGGGACACCACCCTGGTGGCGAGTCCACCAGCAGCAACACAAGGAAACTCCAGCCTCCTTTTATGGCTGAGTTCAGTCATTTTTTTGTGTCTGTGTTCTGTTAGCTCAATCAAGGTTCAAGTAACAAGGCTTCTCAATTGTGGGCAGGTCTGGACTTCAAGGCCATAAAGTAACTTGCTACCGACCCTAGTCGTCTTCTCTAGGCAGAGAGCAGGCAGAGCCGTGGAGATGACCGATTTTGGGACCATAATGGTGAAAGCGACCTCCTAACTGCTAACTGCTAACTGCTCTAGAAAATCCCATGTGCTCTCAGCTAGGGCGAGCCTCTCCTAGGACAGCAAGGATAGGACACAGTGCTGAACTGGCAACCTC**CAAGCCACAGGTTCC**AGTCTGCTGATGGCTGAGCCTCGTTTTCTGGAAGATAGGCTCTTTGCTGGGCTTTCCTGAATACCAAGCTAAGCCGAGCGAGCGCAAGGAACGCTTACAGGACGCGGTTTCCCAGGACTCCGTGCCATGCGTTCCCCGGCACCGCCGCGCATTCCCACTCTCACAGTAGCCCATAGCCGAGAGCGACAGTGCCCTATGCAGTTGGAATGCACACAACGCGTCCTCCCACCCGCACCCGCGATGGGGGCACACTCGGTTCACTCTTCCCGCCTACAGAGGGGTCTGACCCGTAGTCCCAGCAAAGTTAGGGTCACCTTGGCCCACATGCCTTGCATTCAGCAGCCCCACTGCCTATGAGGAGGTTACCACCGATCACTACAGCTTCTCTCCACCTCTGcgcccccacccccatcccacgcgtcacacccccgcccccgccGTTCCCTGTGATGGCCGGCGGCGGGAGAGAGCTGGGGACGGAGCGGGAAATCGGGagaatgaaaaaaataaagaaagaaagaaagaagaagaagaagCGGGAACCCGGAGTCGCCACAGCGTGATGGGAGGCAGAGTCTCTCCTTCCTCCTCCTGGATTCCACTCCAGCCACCCTTCCCGGGAATCTCTTTGGCTATTGCGCCTGCTTAGTGCACGTCCTTTCCAACTTTCTCCCTCCCATCTTCAGATGGAGAAAAAGTGGTCTGGTCCCGGTCTGCAGGTGGAGGGGAGCTGGCGCGAAAAACATACCGTCTGCTGCGGCCACCCCTCCGCGGAAGGCGGTCAAACTCTGGAGCCAAACTCCCAGGGCCACCAGGAGCAGGGCTTTGCTCTCCATCTCCGGGCGCGCCCTTCTGCTTTGCTGCTGGAACTGCGCTGCGCGGGCAGTCAGGCGGGCGGGGATCCGCGTTGCAAAGGGTTGAGCCGGAGGCGTCAGAGGACTAGAGCGCGGTGGAGCGCCGCGGGGCAAGTCAACCTTTAAAGGGGAGGGCAGAACAGCTACAAGGGGCAGAATTTCTTGGAGGAGGAGGAATCGAGTCTGACACCGTTTCCACTGCACAGCTGTTTAAGTGACTGGAAATATGCAAATAAGCCTCATCACCTATTGGCTATAAAATCATAAGTTGGGGGGAGGACTCTAAATTCACTTCCAAATATTTGAGCACACTGTAACGAGGCTCAACGGTGCCAGCGAGAAGAAGGGGAAAGGGCAGGAGGGAAAGCTTGCTTGTCGTTTAGGCTCCAGTTTGCCTATCCAGAGGAACCTGCTCCCCAGGGACCCCTGTAGAGGCCGGGAGGAGGGGTTTACGCTGATTTATGTTAAGTAGTCTACGATCAGTTTTCTGAGGGGAAGAAGTTTTGCTCTTAAACAGCGCGTGTGTCCAACACTTTCACCCCCTCCCTTTTGATAAATCTTTGTTAAACTTGAAAGATCCTTTCAAACGTACCCCCGCCACACACACACATACAGGTCCCCACGGGCCAAAGGGACCTTAGGACACATATTGCTCAGCGCCTGGTGCCTGCACGGTCTGCCTTTTATCGTTGGCAATATGGATCTCCTTCTGTGGTAGAAGAAAGGAAATTAGTTAATAATTCTAATTTTTACCTTATGTAAATTGTACTCGATCCTCTAATTCAGTAAGAAACTCAAGAATAACATCTATTTTCAGAGGCAGAAAGTCATGGTCAAATATTGTAAACTATCCTGGACTTTCAGTGGGAGAAGGCATGCCAAATTACCAATCCAAACTTACAAAGACAGTACATAGATTAGCGATGTTCCAGGGTGAGGGGGATATGGGGCGCTGGGAGTGTGGGGGCAGGGTGAGGATCTTGATGGAAATATCCCGAATTCCACTAGGCTTGTCATTGTATGACTGTGTAGATGCTTTAATATTAAATTGTGCTCTGCCAGGGTTGAGACTGAGCTTTTGCAAAGTGGATTAGCACATGTTTGTAGAAAAGCAAGAAAAATAAAATCACATCGTATACTGTGCTCATCTGCAAGTATCTTTTTAAATGAAGAAGGTGTTGATTAGCTAGAGAAAACCATAAAGATTAAATTCCCCACCTTAATGCTGAGAAGCCGTCTCAAAAAATCAGTTCTCTTTACTGATCTCAGTATTTTTAATATGGTCTCCTCTTcacacacacacacacacacacacacacacacacacacac |

**Table S2.** List of Reagents and Antibodies Used for Experimental Detection.

| Reagent Name | Manufacturer | Dilution ratio |
| --- | --- | --- |
| Paraformaldehyde (PFA) | McLean | 4:100 |
| Pentobarbital sodium | China Pharmaceutical Shanghai Chemical Reagents Company | 1:100 |
| Goat serum for blocking | Zhongshan Jinqiao, China | 1:10 |
| Alexa 488-labelled goat anti-rabbit IgG antibody | Life Technology | 1:600 |
| monoclonal mouse anti-CD31 antibody | Sigma, Japan | 1:400 |
| monoclonal mouse anti-NF200 antibody | Sigma, Japan | 1:400 |
| Goat anti-Rabbit IgG (H+L) Cross-Adsorbed Secondary Antibody Alexa Fluor® 568 | ThermoFisher, USA | 1:300 |
| Alexa Fluor® 488-conjugated AffiniPure Goat Anti-mouse IgG (H+L) | Jackson ImmunoResearch, USA | 1:400 |
| DAPI | Merck, Germany | 2μg/mL |
| Rabbit anti-rat S-100 polyclonal antibody | Boster | 1:100 |
| Alexa 555-labelled goat anti-rabbit IgG antibody | Life Technology | 1:600 |
| Alexa 647-labelled goat anti-rabbit IgG antibody | Life Technology | 1:600 |
| Opti-Men | Gibico, USA |  |
| Runx2-OE / EGFP vector | Cyagen Biosciences, China | MOI=200 |
| SMART-Seq ® HT Kit | Clontech |  |
| Anti-Histone H3 (trimethyl K27) antibody- ChIP grade | Abcam, USA | 1:50 |
| Anti-Runx2 antibody - ChIP grade | Abcam, USA | 1:50 |
| Recombinant anti-CTCF antibody - ChIP grade | Abcam, USA | 1:50 |
| Rabbit Anti-Mouse IgG H&L: | Abcam, USA | 1:100 |
| RevertAid First Strand cDNA Synthesis Kit | Thermo Fisher Scientific |  |
| FastStart Universal SYBR Green Master Mix | Roche, Switzerland |  |
| Lipofectamine3000 Reagent | Thermo, Massachusetts, USA |  |

**Table S3.** Important Equipment Used in the Experiments. Software and Manufacturer List.

| Device/software name (version) | | Manufacturer | |  |
| --- | --- | --- | --- | --- |
| Freezing microtome | | 6 L FREEZONE PLUS, Labconco, USA | |  |
| Stereo microscope | | Carl Zeiss, Germany | |  |
| Laser scanning confocal microscope | | ZESS LSM800,Germany | |  |
| Gene Ontology (GO) | | http://www.geneontology.org | |  |
| Kyoto Encyclopedia of Genes and Genomes Pathway (KEGG) | | http://www.genome.jp/kegg/ | |  |
| Protein Data Bank (PDB) | | https://www.wwpdb.org/ | |  |
| STRING | | https://cn.string-db.org/cgi/input.pl | |  |
| Photoshop CS6 | | Adobe Systems | |  |
| ImageJ | | National Institutes of Health, USA | |  |
| SPSS 20.0 | | IBM, USA | |  |
| GraphPad Prism 9.3 | | GraphPad Software | |  |
| StepOnePlus Real-Time PCR instrument | | Thermo Fisher Scientific | |  |
| Electric constant temperature water bath pot (DK-8D) | | Shanghai Yiheng Technology Co., Ltd | |  |
| Electrophoresis instrument (EPS 300) | | Shanghai Tianneng Technology Co., Ltd. | |  |
| PCR amplifier 2700 | | Applied Biosystems | |  |
| Electronic balance (BS-2000S) | | Beijing Sartorius Instrument Systems Co., Ltd. | |  |
| Constant temperature shaker MTD-8222 | | Jinghong Co., Ltd. | |  |
| Desktop constant temperature shaker SHK-99-Ⅱ | | North Tongzheng Biotechnology | |  |
| Gel imaging system Tanon-1200 | | Shanghai Tianneng Technology Co., Ltd. | |  |
| Microplate reader Molecular Devices SpectraMax M5 | | MD, USA | |  |
| Bioptic Qsep400 Analyzer | Bioptic Inc., China | |  | |
| Qubit and Agilent Bioanalyzer 2100 | Agilent Technologies, USA | |  | |
